# Post-discharge all-cause mortality in COVID-19 recovered patients hospitalized in 2020: the impact of chronic kidney disease

**DOI:** 10.1590/S1678-9946202466001

**Published:** 2024-01-05

**Authors:** Guilherme Schittine Bezerra Lomba, Pedro Henrique Abreu da Silva, Natalia Fonseca do Rosário, Thalia Medeiros, Lilian Santos Alves, Andrea Alice Silva, Jorge Reis Almeida, Jocemir Ronaldo Lugon

**Affiliations:** 1 Universidade Federal Fluminense, Faculdade de Medicina, Niterói, Rio de Janeiro, Brazil; 2 Universidade Federal Fluminense, Faculdade de Medicina, Laboratório Multiusuário de Apoio à Pesquisa em Nefrologia e Ciências Médicas, Niterói, Rio de Janeiro, Brazil; 3 Universidade Federal Fluminense, Faculdade de Medicina, Departamento de Patologia, Niterói, Rio de Janeiro, Brazil; 4 Universidade Federal Fluminense, Faculdade de Medicina, Departamento de Medicina Clínica, Divisão de Nefrologia, Niterói, Rio de Janeiro, Brazil

**Keywords:** COVID-19, Mortality, Chronic kidney disease, Hospitalization, Discharge

## Abstract

In Brazil, the COVID-19 burden was substantial, and risk factors associated with higher in-hospital mortality rates have been extensively studied. However, information on short-term all-cause mortality and the factors associated with death in patients who survived the hospitalization period of acute SARS-CoV-2 infection is limited. We analyzed the six-month post-hospitalization mortality rate and possible risk factors of COVID-19 patients in a single center in Brazil. This is a retrospective cohort study focused on a six-month follow-up. The exclusion criteria were death during hospitalization, transference to another hospital, and age under 18. We collected data from the charts of all hospitalized patients from March 2020 to December 2020 with a positive RT-PCR test for SARS-CoV-2, resulting in a sample size of 106 patients. The main outcome was death after hospitalization, whereas comorbidities and demographics were evaluated as risk factors. The crude post-hospitalization death rate was 16%. The first 30 days of follow-up had the highest mortality rate. In a Cox regression model for post-hospitalization mortality, previous chronic kidney disease (HR, 4.06, 95%CI 1.46 – 11.30) and longer hospital stay (HR 1.01, 95%CI 1.00 – 1.02) were the only factors statistically associated with death. In conclusion, a high six-month all-cause mortality was observed. Within the six-month follow-up, a higher risk of death was observed for patients who had prior CKD and longer hospital stay. These findings highlight the importance of more intensive medical surveillance during this period.

## INTRODUCTION

As of September 2022, the number of deaths related to COVID-19 pandemic reached nearly six million worldwide, posing an unprecedented challenge to public healthcare systems and clinical practice^1^. In Brazil, the COVID-19 burden was substantial, and related casualties in the same period reached 690,000^2^. The disease pathophysiology and risk factors associated with higher in-hospital mortality have been extensively studied to improve its clinical management and to identify patients with poor prognosis at early stages^3^.

In this setting, the risk factors associated with a complicated hospitalization have been well established. However, information on short-term all-cause mortality and its related factors in patients who survived the hospital phase of acute SARS-CoV-2 infection is limited. A case cohort study addressing this topic in patients aged under 60 years reported that all-cause mortality was higher within the first five weeks of follow-up^4^.

This study aimed to analyze the six-month post-hospitalization mortality rate of patients with COVID-19 admitted to a single center in Brazil from March 2020 to December 2020.

## MATERIALS AND METHODS

This is a retrospective cohort study performed with data from a third-level university Brazilian hospital. The study was approved by the Ethical Committee of the Medical School of Universidade Federal Fluminense under the Nº 59213722.5.0000.5243. All activities were performed after the patient’s consent was obtained.

Data were collected from the charts of all the patients who were hospitalized from March 2020 to December 2020 with a positive RT-PCR test for SARS-CoV-2. This time frame was selected since vaccination against the infection was not available and only the wild-type virus had been identified in Brazil^5,6^.

This study adopted the following exclusion criteria: (i) death during hospitalization; (ii) transference to another hospital; and (iii) age under 18. The studied variables comprised sex, skin color, age, comorbidities, admission to an intensive care unit (ICU), and length of hospital stay (LOS). Other factors computed were the need for orotracheal intubation (OTI) and mechanical ventilation (MV), acute kidney injury (AKI) requiring kidney replacement therapy (KRT), and intravenous catecholamines administration. The comorbidities of interest included diabetes, hypertension, obesity, current or past neoplasia, asthma/chronic obstructive pulmonary disease (COPD), current immunosuppressive therapy, chronic kidney disease (CKD), and stroke. Diabetes, hypertension, and COPD diagnoses were obtained according to medical history. Obesity was phenotypically defined by the assistant physician. CKD was defined by a glomerular filtration rate (GFR) below 60 ml/min/1.73m^2^ at admission (as estimated by the 2021 CKD-EPI creatinine equation). The stage of CKD was determined following the KDIGO guidelines.

For the six-month follow-up, the mortality rates after hospital discharge were analyzed. Patients’ status after discharge was obtained by searching the Court of Justice of the State of Rio de Janeiro birth and death certificate platform^7^.

### Statistical analysis

Continuous data are reported as mean ± standard deviation (SD) and categorical variables as frequencies. Differences in continuous data between alive and deceased participants were analyzed using unpaired T test or Mann-Whitney test, and differences between frequencies by the Chi-squared or the Fisher test as appropriate.

Time to death was analyzed by the Kaplan-Meier method. Associations with death were tested by Cox proportional Hazard regression modeling. Only variables with a high probability of association with the outcome in the univariate analysis (P<0.10) were included in the multivariate models, except for age, which was forced into the model. Significance was set at P<0.05.

Statistical analyses were performed in R 4.1.0 and SPSS programs, version 18.0 (IBM, USA).

## RESULTS

**Figure 1:**
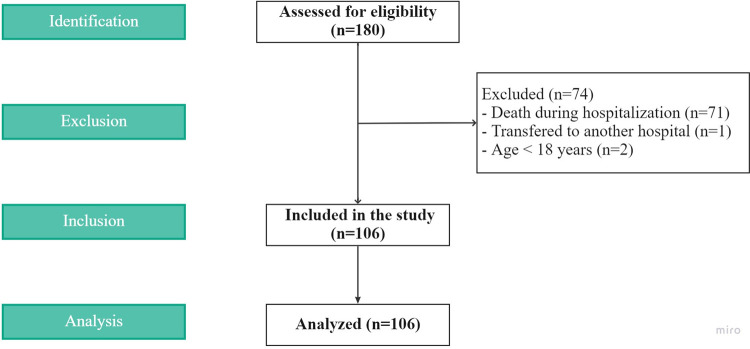
Patient flow diagram.

**Figure 2:**
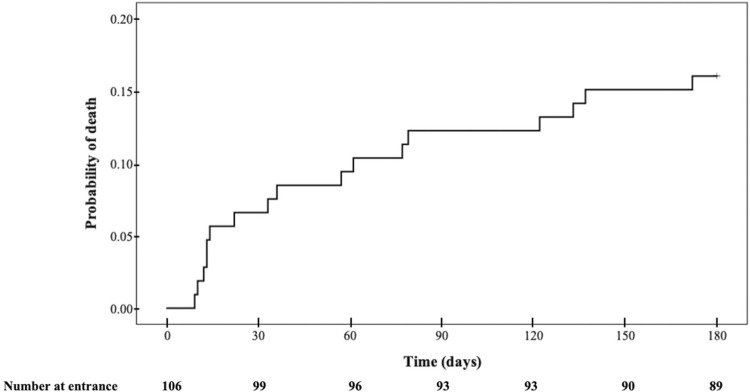
Cumulative probability of death after COVID-19 hospitalization.

A total of 180 patients were initially suitable for eligibility. After exclusion criteria, 106 participants were included in this study (Figure 1). Table 1 shows the general characteristics of the participants, further categorized by patient outcome at the six-month follow-up. We highlight that, out of the 15 patients with CKD, two were in stage G3a, two in G3b, four in G4, and seven in G5 (three of whom were on chronic renal replacement therapy). We identified 17 deaths throughout the six-month follow-up, resulting in a cumulative probability of death of 16.0% (Figure 2). In a Cox regression model for six-month post-hospitalization mortality, CKD (hazard ratio, HR, 4.06, 95%CI 1.46 – 11.30) and hospital LOS (HR 1.01, 95%CI 1.00 – 1.02) were associated with a higher risk of death (Table 2).

**Table 1:** General characteristics of the studied population, further categorized by patient outcome at the six-month follow-up (data are shown as number and percent unless specified).

	Overall (N = 106)	Death within 6 months (N = 17)	Alive at 6 months (N = 89)
Male sex	56 (52.8)	9 (52.9)	47 (52.8)
Age^a^ (years)	58 ± 16	63 ± 9	58 ± 17
Diabetes	31 (29.2)	4 (23.5)	27 (30.3)
Hypertension	71 (67.0)	14 (82.4)	57 (64.0)
Obesity	19 (17.9)	0(0.0)	19 (21.3)
Neoplasia^b^	40 (37.7)	8 (47.1)	32 (36.0)
Asthma/COPD	19 (17.9)	1 (5.9)	18 (20.2)
Immunosuppression^c^	25 (23.6)	4 (23.5)	21 (23.6)
CKD	15 (14.2)	6 (35.3)	9 (10.1)
G3a	2	1	1
G3b	2	1	1
G4	4	1	3
G5 on CT	4	2	2
G5 on chronic RRT	3	1	2
Stroke	6 (5.7)	2 (11.8)	4 (4.5)
ICU Admission	40 (37.7)	5 (29.4)	35 (39.3)
Orotracheal intubation	18 (17.0)	3 (17.6)	15 (16.9)
Onset of RRT on ICU	12 (11.3)	2 (11.8)	10 (11.2)
With previous CKD	5	2	3
Without previous CKD	7	0	7
IV catecholamines infusion	15 (14.2)	4 (23.5)	11 (12.4)
Number of failed organs			
0	81 (76.4)	13 (76.5)	68 (76.4)
≥1	25 (23.6)	4 (23.5)	21 (23.6)
Hospital LOS^a^ (days)	29 ± 34	46 ± 47	24 ± 26

^a^mean ± SD; ^b^current or past; ^c^current or within the past 3 months; COPD = chronic pulmonary obstructive disease; CKD = chronic kidney disease; CT *=* conservative treatment; RRT *=* renal replacement therapy; ICU = intensive care unit; LOS = length of stay.

**Table 2:** Univariate and multivariate Cox proportional hazards regression analysis for risk factors associated with mortality up to 180 days after hospital discharge.

	Univariate		Multivariate	

	HR (95% CI)	P	HR (95% CI)	P
Male sex	1.20 (0.27 – 5.36)	0.811	-	-
Age, years	1.02 (0.99 – 1.05)	0.228	1.02 (0.99 – 1.06)	0.203
Comorbidities				
Diabetes	0.73 (0.24 – 2.25)	0.589	-	-
Hypertension	2.43 (0.70 – 8.44)	0.164	-	-
Obesity	0.04 (0.00 – 6.29)	0.206	-	-
Neoplasia^a^	0.97 (0.32 – 2.97)	0.958	-	-
Asthma/COPD	0.26 (0.03 – 1.99)	0.197	-	-
Immunosuppression^b^	1.33 (0.34 – 5.15)	0.565	-	-
CKD	4.16 (1.53 – 11.26)	0.005	4.06 (1.46 – 11.30)	0.007*
Stroke	2.73 (0.62 – 11.99)	0.182	-	
ICU admission	0.66 (0.23 – 1.88)	0.437	-	-
Orotracheal intubation	1.02 (0.29 – 3.54)	0.978	-	-
Hemodialysis				
AKI-related	1.07 (0.25 – 11.41)	0.925	-	-
IV catecholamines infusion	2.54 (0.49 – 4.70)	0.264	-	-
Number of failed organs				
0	Reference			
≥ 1	1.15 (0.70 – 1.89)	0.591	-	-
Hospital LOS	1.02 (1.01 – 1.03)	0.003	1.01 (1.00 – 1.02)	0.008*

^a^current or past; ^b^current or within the past 3 months; COPD = chronic pulmonary obstructive disease; CKD = chronic kidney disease; ICU = intensive care unit; LOS = length of stay.

## DISCUSSION

Several studies have investigated the short-term consequences of COVID-19 infection. This study analyzed the six-month all-cause mortality of hospitalized COVID-19 patients in a single-center in Brazil. The most relevant findings in this study are the high six-month all-cause mortality (16.0%) and the identification of previous CKD and long hospital LOS as risk factors for death during the first six months after hospitalization.

Findings of studies addressing post-discharge all-cause mortality in recovered COVID-19 patients are inconsistent. While a recent meta-analysis showed an average mortality rate of 7.6% at one-year after recovery, others reported rates as high as 20%, which corroborates our results^8-11^. This discrepancy may be partially due to heterogeneity among patients’ comorbidities and COVID-19 severity^11^. Interestingly, the studies that analyzed more severe cases have presented numbers closer to ours^12,13^. In fact, our sample only included patients that were already registered and being treated in our unit, a tertiary care teaching hospital, favoring the inclusion of participants with a high likelihood of having comorbidities.

We highlight that study participants were not vaccinated against SARS-CoV-2 at the time of admission since vaccination against COVID-19 in Brazil began on January 17, 2021^6^. Moreover, they were most likely infected with the wild-type virus, as the cohort was constrained from March to December 2020, an epidemiological period corresponding to the first SARS-CoV-2 Brazilian wave^5^.

Consistent with the findings of our study (Figure 2), most deaths in large American, European, and Asian cohorts occurred during the first 30 days of follow-up^4,8,11^. These findings indicate that recovered COVID-19 patients may be particularly susceptible to fatal events in the short-term period post-discharge. Whether the decrease in life expectancy is related to the COVID-19 infection itself or to the worsening of underlying comorbidities is still uncertain^14^.

In this study, preexistent CKD and hospital LOS were identified as risk factors for post-discharge mortality within the six months following discharge. Surprisingly, CKD was not identified as a risk factor for short-term mortality post-discharge in prior studies^14^, making this study the first to report this association. However, CKD has been associated with high incidence of COVID-19 and increased in-hospital all-cause mortality^15,16^. Indeed, CKD is recognized as a major risk factor for increased hospitalization and mortality during hospitalization, both in several clinical and surgical circumstances^17-19^. Other studies suggest that patients with kidney failure develop a more severe course of COVID-19; the fatality rate of Brazilian hemodialysis patients was found to be seven times higher than the general Brazilian population affected by COVID-19^20,21^. Thus, the finding of CKD as a risk factor for post-discharge short-term mortality in recovered COVID-19 patients is consistent with previous knowledge.

CKD pathophysiology includes premature immunological aging, which contributes to a proinflammatory state that can predispose to infections^22,23^. CKD may also be associated with an increased cardiovascular risk and greater frailty^23-26^. Notably, during the analyzed period, access to treatment and invasive interventions for medical emergencies was hampered by the pandemic, increasing the potential risk offered by the presence of comorbidities^14,27,28^. Although the etiology of the CKD in the present study was not determined, we highlight that 33.7% and 86.7% of our CKD patients also had diabetes and hypertension, respectively, the most common causes of CKD worldwide.

A long hospital LOS was also associated with an increased risk of post-discharge mortality. Longer LOS is a known risk factor for overall short-term complications such as early readmissions and higher mortality rates in several scenarios^29^. Surprisingly, a recent meta-analysis addressing such issues did not mention the role of LOS in the post-discharge short-term events in surviving COVID-19 patients^1^. A long hospital LOS could be a consequence of underlying comorbidities at admission or COVID-19 severity, conditions that could impact the post-discharge short-term mortality^30-32^.

To assess the robustness of our findings, we conducted a sensitivity analysis by constructing a Cox Regression model in which we forcefully entered hypertension, diabetes mellitus, and obesity. Reinforcing our findings, only CKD (HR 3.31, 95%CI 1.16 – 9.51) and hospital LOS (HR 1.01, 95%CI 1.00 – 1.02) were significantly associated with post-discharge mortality.

This study has some limitations. It dealt with a small sample derived from a single center and presented the inherent bias of its retrospective design. In addition, our sample presented a high rate of comorbidities since data were derived from a tertiary care teaching hospital. Additionally, the study lacks a control group, which we sought to overcome by comparing our findings with those of previous publications. Finally, we restricted the follow-up to 6 months, considering that the low number of remaining patients could prevent reliable conclusions. However, these limitations do not invalidate our findings, which contribute to filling important knowledge gaps on post-hospitalization mortality of Brazilian patients that recovered from COVID-19.

## CONCLUSION

In summary, in this single-center study, we observed a high six-month all-cause mortality of COVID-19 patients who survived hospitalization. The first 30 days of follow-up presented the highest mortality rates. Within our follow-up period, patients who had prior CKD and longer LOS exhibited a higher risk of death. These findings suggest that more intensive medical surveillance during this period could help mitigate these outcomes. Further studies are necessary to better substantiate our findings.
